# Type 1 diabetes in North East England and North Cumbria: patterns and time trends in 0–14-year-olds from 2012 to 2020

**DOI:** 10.3389/fpubh.2023.1193403

**Published:** 2023-08-11

**Authors:** Louise Hayes, Tim Cheetham, Colin Muirhead, Neil Hopper, Judith Reid, William Lamb, Jenny Foster, Richard J. Q. McNally

**Affiliations:** ^1^Population Health Sciences and Translational and Clinical Research Institutes, Newcastle University, Newcastle upon Tyne, United Kingdom; ^2^North East England and North Cumbria Diabetes Network, Sunderland, United Kingdom

**Keywords:** type 1 diabetes, children, patterns, time trends, EPI—epidemiology

## Abstract

**Introduction:**

It is important to understand patterns in the epidemiology of type 1 diabetes because they may provide insight into its etiology. We examined the incidence of type 1 diabetes in children aged 0–14 years, and patient demographics and clinical parameters at presentation, over the period 2012–2020 using the North East and North Cumbria Young Persons diabetes register.

**Methods:**

Patients up to the age of 14 years with type 1 diabetes, and their families- managed in a total of 18 young persons diabetes clinics—were approached in person at the time of clinic appointments or in the days following diagnosis and they consented to their data being included in the register. Data were submitted regionally to a central unit. Descriptive statistics including crude and age-specific incidence rates were calculated. Temporal trends were analyzed using Joinpoint regression. Comparisons in incidence rates were made between age, sex and areas of higher and lower affluence as measured by the Index of Multiple Deprivation (IMD).

**Results:**

A total of 943 cases were recorded between January 2012 and December 2020. Median age at diagnosis was 8.8 years (Q1: 5.3, Q3: 11.7). There were more males than females (54% male). The median HbA1c at diagnosis was 100 mmoL/L (IQR: 39) and over one third (35%) were in ketoacidosis (pH < 7.3). Crude incidence decreased from 25.5 (95% confidence interval [CI] 20.9, 29.9) in 2012 to 16.6 (95% CI: 13.0, 20.2) per 100,000 in 2020 (5.1% per annum, 95% CI 1.1, 8.8%). During the period of the study there was no evidence of any trends in median age, HbA1c, BMI or birthweight (*p* = 0.18, 0.80, 0.69, 0.32) at diagnosis. Higher rates were observed in males aged 10–14 years, but similar rates were found for both sexes aged 0–9 years and there was no difference between areas of higher or lower deprivation (*p* = 0.22).

**Conclusion:**

The incidence of diabetes in the young may be falling in the North East of England and North Cumbria. The reasons are unclear as there were no associations identified between levels of deprivation or anthropometric measurements. Potential mechanisms include alterations in socioeconomic background or growth pattern. Further research is needed to understand the reasons behind this finding.

## Background

An increase in the incidence and prevalence of diabetes in children across Europe has been observed over recent decades (1–3). While there has been a suggestion of a plateauing of incidence in some nations (4, 5), the annual rate of increase in incidence in Europe has been approximately 3% with a higher increase (4.8%) being seen in children aged 0–4 years than in older children (1, 3). The incidence has doubled over a period of 20 years and as such represents an increasing burden to families and health services.

The etiology of pediatric diabetes is not fully understood. Existing evidence suggests a complex process with both genetic and environmental factors implicated (6–8). A cyclical pattern of incidence has also been observed in some parts of Europe including parts of England, although the mechanism for this and its implications in terms of disease development is unclear (3, 9).

Registry data from areas of the UK such as Yorkshire, Oxford, Scotland and South Wales have shown that case data can refine the planning and monitoring of health services. Registries can also provide insights into disease etiology and putative risk factors (10, 11). The potential value of establishing national pediatric diabetes registries in all four UK countries has been highlighted (12).

The Diabetes Network in the North East of England and North Cumbria began to establish a regional register in 2010 with comprehensive data collected from 2011. The North East and North Cumbria region extends from the Scottish border in the north, to the Tees area in the south and to the west coast of North Cumbria, covering a population of 2,924,000, of whom 589,280 were aged 0–18 years according with ONS 2011.^1^ The population in the region is predominantly of white ethnicity (95.3%).^2^ Some of the most economically deprived local authority districts in the country are within the North East of England and North Cumbria region.^3^

The aims of this study were: to establish if type 1 diabetes incidence in children and young adults aged 0–14 years in the North East of England and North Cumbria is changing over time, to look for potential associations between diagnosis of type 1 diabetes and age, sex, deprivation level and patient characteristics at diagnosis.

## Methods

### Identification of cases

The North East and North Cumbria region has an active group of interested health professionals with representation from all of the 14 young persons diabetes teams in the locality. The teams of the regional units were contacted in 2010 and they agreed to obtain consent from known and new patients with diabetes. What constituted pertinent information and hence data fields was agreed at regional meetings and by the network leads. Units consented families and collected data and thus provided a comprehensive dataset for each local clinic. Type 1 diabetes was defined as the diagnosis given by the consultant in charge of the case, dependence on insulin from diagnosis and/or proneness to ketosis. Definitions of type 1, type 2, neonatal and genetic diabetes followed National Institute for Health and Care Excellence Guidelines (NICE).^4^

### Consent/assent

Patients with diabetes and their families were approached in person at the time of clinic appointments or in the days following diagnosis. Families were provided with an information sheet and given time to decide whether they would like to take part prior to completing the relevant consent or assent forms. All children under the age of 16 years are managed in a children’s diabetes service.

### Ethical approval

Ethical approval for the registry was given by the research ethics committee (NRES Committee North East—Newcastle and North Tyneside 1) on 10th February 2017 (REC reference number: 17/NE/0011) and renewal was granted on 6th June 2022 (REC reference number: 22/NE/0061).

### Population data

Population denominator data were obtained from the Office for National Statistics for the area covered by the register, by age, sex and single year. Crude incidence, by year, was calculated for all 0–14-year olds diagnosed with Type 1 diabetes in the North East and North Cumbria, and by sex using mid-year population estimates for the years 2012 to 2020. Age group (0–4, 5–9 and 10–14 years, where 0–4 years includes children aged up to 1 day less than 5 years, 5–9 years includes children aged from 5 years to 1 day less than 10 years, 10–14 years includes children aged from 10 years up to 1 day less than 15 years) and sex specific rates were also calculated.

### Demographic data

Area of deprivation was measured using the Index of Multiple Deprivation (IMD),^5^ grouped into quintiles based on the distribution across England, with quintile 5 representing the most affluent areas and quintile 1 areas with the highest level of deprivation. This area-based measure was used as a proxy for individual level deprivation (as this was not possible to obtain).

### Statistical analysis

**Figure 1:**
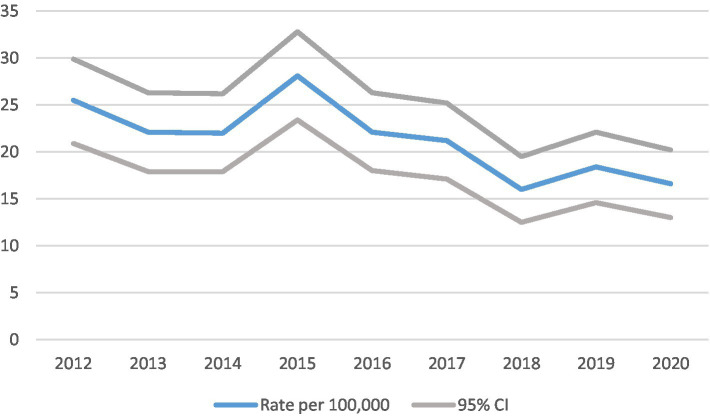
Cases and crude incidence rate of type 1 diabetes in North East England in 0–14 year olds, by year, diagnosed 2012–2020 (*n* = 943).

**Figure 2:**
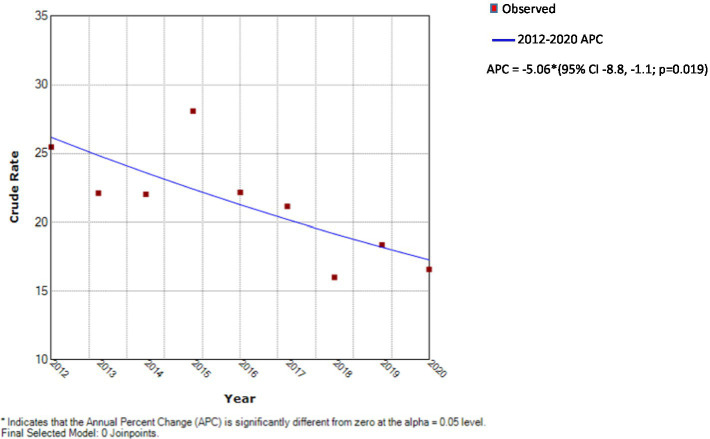
Results of Joinpoint analysis—crude incidence rates per 100,000 by year.

Sex, ethnicity, IMD quintiles and diabetic ketoacidosis (DKA) were summarized using counts and percentages, while age, body mass index (BMI), birthweight, glycated hemoglobin (HbA1c) and PH were described using medians, lower and upper quartiles (Tables 1, 2). The Kruskal-Wallis H test was used to test for differences in age, BMI, birthweight and HbA1c between years (Table 2). Crude incidence rates per 100,000 population, together with 95% confidence intervals were calculated for the entire study period and separately for each of the years from 2012 to 2020 (Figure 1). Joinpoint regression was used to analyze temporal trends in the incidence rate (Figure 2). Rates were calculated by age group (0–4, 5–9, 10–14 years) and separately for males and females. Comparisons between males and females were facilitated through calculation of rate ratios (Table 3). Crude incidence rates were calculated by deprivation quintile. Comparisons were made between each of the four most affluent quintiles and the most deprived quintile using rate ratios (Table 4). In addition, the trends in rates with deprivation quintile were analyzed using a Mann-Kendall test. Analyses were conducted using Stata Version 16. The critical value used to define statistical significance was taken to be *p* < 0.05.

**Table 1:** Characteristics of 943 children aged 0–14 years diagnosed with type 1 diabetes in North East England, 2012–2020.

Characteristic		
Sex (*n; %*)		
Male	506	53.7
Female	434	46.3
Age—years *(median; Q1, Q3)*	8.8	5.3, 11.7
Ethnicity (*n; %*)		
White	864	91.6
South Asian	11	1.2
Black	4	0.4
Mixed race	14	1.5
Other	6	0.6
Not stated	44	4.7
IMD quintile (*n; %*)		
(most deprived) 1	316	34.9
2	209	23.1
3	123	13.6
4	129	14.3
5	128	14.1
BMI at diagnosis *(median; Q1, Q3)*	16.2	14.8, 18.5
Birthweight—g *(median; Q1, Q3)*	3,409	3,010, 3,742
HbA1c *(median; Q1, Q3)*	100	80, 119
PH *(median; Q1, Q3)*	7.35	7.24, 7.39
DKA (PH ≤ 7.3) *(n; %)*	217	35.1

*IMD, *n* = 905; BMI, *n* = 354; Birthweight, *n* = 745; HbA1c, *n* = 875; PH, *n* = 585.

**Table 2:** HbA1c and BMI at diagnosis and birthweight by year of diagnosis; medians (Q1, Q3).

	Age	HbA1c	BMI	Birthweight—g
2012	*n* = 123 8.02 (4.97, 11.42)	*n* = 88 100 (83.5, 119.5)	*n* = 32 15.9 (14.9, 18.3)	*n* = 95 3,490 (3,170, 3,750)
2013	*n* = 107 9.02 (5.49, 12.03)	*n* = 75 102 (79, 120)	*n* = 45 15.2 (16.2, 17.1)	*n* = 84 3,435 (2,948. 3,824)
2014	*n* = 107 8.74 (5.23, 10.77)	*n* = 74 97.5 (81, 122)	*n* = 47 16.7 (14.8, 19.1)	*n* = 82 3,370 (3,005, 3,770)
2015	*n* = 137 8.81 (5.66, 11.02)	*n* = 112 97 (77, 115.5)	*n* = 53 15.7 (14.5, 18.2)	*n* = 99 3,487 (3,130, 3,742)
2016	*n* = 109 8.60 (5.25, 11.70)	*n* = 91 101 (78, 120)	*n* = 49 16.0 (14.5, 18.7)	*n* = 85 3,454 (3,062, 3,800)
2017	*n* = 105 9.35 (6.23, 12.21)	*n* = 92 103 (80, 120.5)	*n* = 37 16.8 (15.0, 17.8)	*n* = 81 3,350 (3,061, 3,710)
2018	*n* = 80 7.74 (3.90, 11.52)	*n* = 68 104.5 (83.5, 120)	*n* = 39 16.6 (14.7, 18.1)	*n* = 68 3,215 (2,837, 3,651)
2019	*n* = 91 9.62 (6.27, 11.75)	*n* = 87 98 (82, 126)	*n* = 32 16.9 (15.3, 19.6)	*n* = 81 3,425 (3,000, 3,719)
2020	*n* = 83 8.25 (6.36, 11.58)	*n* = 79 101 (85, 118)	*n* = 20 15.7 (14.6, 19.0)	*n* = 70 3,280 (2,860, 3,740)
*p*-value for trend*	0.18	0.80	0.69	0.32

*Kruskal-Wallis H test.

**Table 3:** Cases and age specific rates of type 1 diabetes in North East England by age and sex*, in 0–14 year olds, diagnosed 2012–2020 (*n* = 943).

	n	Rate per 100,000	(95% CI)	Rate ratio (95% CI)**
Male and female				
0–4 years	204	13.8	(11.9, 15.7)	0.94 (0.71, 1.24)
5–9 years	374	24.6	(22.1, 27.1)	1.05 (0.86, 1.29)
10–14 years	362	25.2	(22.6, 27.8)	0.76 (0.61, 0.93)
**Total**	**940**	**21.3**	**(19.9, 22.7)**	**0.91 (0.80, 1.03)**
Male				
0–4 years	108	14.2	(11.6, 16.9)	
5–9 years	187	24.0	(20.6, 27.4)	
10–14 years	211	28.6	(24.7, 32.5)	
**Total**	**506**	**22.2**	**(20.3, 24.2)**	
Female				
0–4 years	96	13.4	(10.7, 16.1)	
5–9 years	187	25.3	(21.7, 28.9)	
10–14 years	151	21.6	(18.2, 25.0)	
**Total**	**434**	**20.1**	**(18.3, 22.0)**	

*Sex missing for 3 cases.

**Rate ratio for female to male cases diagnosed.

Bold value means total (0–14 years).

**Table 4:** Cases and crude incidence rates of type 1 diabetes in North East England by deprivation*, in 0–14 year olds, diagnosed 2012–2020 (*n* = 905).

IMD quintile	n	Rate per 100,000	(95% CI)	Rate ratio (95% CI)**
(most deprived) 1	316	18.5	(18.3, 18.7)	1.00
2	209	20.2	(19.9, 20.5)	1.09 (0.92, 1.30)
3	123	19.5	(19.2, 19.9)	1.06 (0.86, 1.30)
4	129	23.0	(22.6, 23.4)	1.24 (1.01, 1.53)
5	128	22.3	(21.9, 22.8)	1.21 (0.98, 1.48)
p for trend***				0.22

*IMD: index of multiple deprivation, quintiles for England.

**Rate ratio for each quintile of IMD compared to most deprived quintile.

***p-value from Mann Kendall test for trend.

## Results

Population characteristics (Table 1).

Prospective data collection began in January 2012 and the analysis presented here includes 943 cases recorded in 0–14 year olds from January 2012 to December 2020. Median (quartile 1, quartile 3) age at diagnosis was 8.8 (5.3, 11.7). The majority of young people with diabetes were male (54%) and 92% were of white ethnicity (Table 1). Over a third of patients on the registry lived in areas in the most deprived quintile of socio-economic deprivation. Median (Q1, Q3) BMI at diagnosis was 16.2 (14.8, 18.5). Median (Q1, Q3) birthweight was 3,409 g (3,010 g, 3,742 g). Median (Q1, Q3) HbA1c was 100 (80, 119). Median (Q1, Q3) PH was 7.35 (7.24, 7.39). The number diagnosed with DKA was 217 (35%).

Birthweight, physical and biochemical characteristics at diagnosis (Table 2).

There was no evidence of a change in HbA1c concentrations (trend test: *p* = 0.80) or BMI at diagnosis (trend test: *p* = 0.69) or of a change in the birthweights (trend test: *p* = 0.32) of children and young people diagnosed with type 1 diabetes over the time period studied.

Trends in incidence rates (Figures 1, 2).

The crude incidence rate of type 1 diabetes fell with time as illustrated in Table 3 and Figure 1. Between 2012 and 2020 (a 9 year period), the incidence fell by approximately one third from 25.5 (95% CI 20.9, 29.9) to 16.6 (13.0, 20.2) per 100,000 children per year. Joinpoint regression analysis indicated that there was a statistically significant annual decrease in incidence of 5.1 (95% CI 1.1, 8.8%, *p* = 0.019) during the period 2012–2020.

Age and sex distribution (Table 3).

There was no evidence for any change in the median age over the study period (trend test: *p* = 0.18). Crude incidence was lower in females than males (RR 0.91; 95% CI 0.80, 1.03), but this difference was statistically significant only in the 10–14 years age group (0.76; 0.61, 0.93) (Table 4). Peak incidence rate was in patients diagnosed at 10–14 years of age with a rate of 25.2 (95% CI 22.6, 27.8) per 100,000 children per year.

Distribution by quintile of deprivation (Tables 4, 5).

**Table 5:** Proportion of cases of type 1 diabetes by IMD quintile in NPDA, NPDA-NENC, and North East and North Cumbria registry.

	NPDA	NPDA—NENC	NE registry
Most deprived	23%	34.3%	34.9%
Second most deprived	20.3%	22.1%	23.1%
Third least deprived	19.1%	15.2%	13.6%
Second least deprived	18.6%	14.5%	14.3%
Least deprived	19.0%	13.9%	14.1%

NPDA, National Pediatric Diabetes Audit; NPDA-NENC, North East and North Cumbria region of NPDA; NE registry, North East and North Cumbria Diabetes Network Registry.

57.6% of patients were in the two most deprived quintiles of IMD. Although diabetes appeared to develop most commonly in families in the fourth IMD quintile, there was no evidence of a trend in rates with deprivation quintile (Mann-Kendall test, *p* = 0.22). The proportions of cases of diabetes in the registry by IMD quintile were similar to the National Pediatric Diabetes Audit – North East and North Cumbria (NPDA-NENC).

## Discussion

The incidence of type 1 diabetes in people aged 14 years and younger appears to be falling in the North East of England and North Cumbria. This pattern of an absence of a recent increase in incidence is not unique and has been described in other parts of the world as well. Studies from Germany, Sweden, the Czech Republic and USA have shown plateaus in incidence, while one study from Finland demonstrated a decrease in incidence (13–17). A large majority of young people with diabetes in the region (approximately 99%) have type 1 diabetes with approximately 1% deemed to have type 2 diabetes and only 0.1% deemed to have monogenic forms and 0.1% cystic fibrosis related diabetes. The nature of the data submitted and the fact that categorizing cases into type 1 or type 2 is not always straightforward means that we cannot be certain that we have captured every single newly diagnosed patient with type 1 diabetes, although we suspect that this is an accurate representation of current trends from 2012 to 2020. We were aware of the potential for young people aged 16 years and above to be seen by adult diabetes teams but we are confident that all cases in the age-range included in this study were seen exclusively by local teams who were actively contributing to the register. The low number of type 2 diabetes cases in part reflects the ethnic mix of the locality which is mostly white, comprising 92% of cases. However, it is worth noting that type 2 diabetes is observed in all ethnic groups, with wide differences in prevalence between ethnicities, particularly in those aged less than 40 years (18). We do not believe that any recent changes in the distribution of ethnicity within the region could account for the marked change in incidence.

A number of environmental factors have been linked to diabetes incidence including variations in the levels of exposure to infectious agents and changes in growth pattern and obesity (8, 19). Associations between the rate of increase in incidence and previous incidence have also been noted with a more rapid increase in countries where type 1 diabetes was previously less common (5). Hence there are a number of potential underlying trends that can impact the number of new cases within a particular locality. The striking feature of our data is the markedly high levels of deprivation, both in absolute terms and also when compared to other parts of England. The levels of deprivation from our registry study were similar to those obtained from the regional national audit.^6^ In our region, 56.4% were in the two most deprived quintiles compared with 43.3% in the whole of England (Table 5). Some researchers have suggested that deprivation can be relatively protective in the context of type 1 diabetes development (5, 20, 21), and one potential explanation for our observations is that the falling incidence, at least in part, may reflect changes in socioeconomic patterning in the region. It should also be acknowledged that falling rates may reflect decreases in the level of ascertainment of cases over time. However, a consistent methodology has been used for data collection during the study period, suggesting that this is unlikely to be a plausible explanation.

### Limitations

As with any registry, we have been keen to ensure that ascertainment is optimized. Our region is characterized by a strong network of professionals and there has been a lot of interest in the development of this registry. There is no good evidence to suggest that ascertainment has changed with time and the falling incidence does not match any subjective patterns that we have observed. Indeed, we were reassured to see that many characteristics of our population are in keeping with other studies in young people with type 1 diabetes, with males more likely to be affected than females and with around one third in ketoacidosis at the time of diagnosis (22, 23). We were also reassured to see that the data on deprivation obtained from the register was similar to the regional audit. It should be noted that although the majority of our period of data collection precedes the onset of COVID this could potentially have impacted some of the variables that we were collecting during the final year of our study period (24).

## Conclusion

We have found novel evidence for a decrease in the incidence of diabetes (preliminary type 1) in children resident in the northern region of England. Only one other recent study from Finland has found a decrease in incidence. There was no evidence for any trend in the association with deprivation, nor were there any changes in anthropometric measurements. The statistical analyses were based on high quality and complete data from a population-based registry. These findings may reflect recent changes in the patterns of exposure to, as yet unidentified, risk factors, or changes in lifestyle.

## Research in context

### What is already known about this subject?

An increase in the incidence and prevalence of diabetes in children across Europe has been observed over recent decades.

### What is the key question?

Has there been a recent increase or decrease in the incidence of type 1 diabetes in the North East of England and North Cumbria?

### What are the new findings?

There has been a notable decrease in the incidence of type 1 diabetes in the North East of England and North Cumbria.

### How may this impact clinical practice in the foreseeable future?

These findings may reflect recent changes in the patterns of exposure to, as yet unidentified, risk factors, or changes in lifestyle. It is important to understand patterns in the epidemiology of type 1 diabetes because they may provide insight into its etiology and influence health care service provision.

## Data availability statement

The datasets presented in this article are not readily available because only non-identifiable data are available. Requests to access the datasets should be directed to Richard.McNally@newcastle.ac.uk.

## Ethics statement

The studies involving human participants were reviewed and approved by the NRES Committee North East—Newcastle and North Tyneside 1. Written informed consent to participate in this study was provided by the participants’ legal guardian/next of kin.

## Author contributions

RM, LH, and TC: design of the study. TC, NH, JR, WL, and JF: collection of data. LH, CM, and RM: analysis of data. All authors: interpretation of data and writing the report.

## Funding

Childhood diabetes registries at Newcastle University have been funded by the North East England and North Cumbria, North West England and East Midlands Diabetes Networks.

## Conflict of interest

The authors declare that the research was conducted in the absence of any commercial or financial relationships that could be construed as a potential conflict of interest.

## Publisher’s note

All claims expressed in this article are solely those of the authors and do not necessarily represent those of their affiliated organizations, or those of the publisher, the editors and the reviewers. Any product that may be evaluated in this article, or claim that may be made by its manufacturer, is not guaranteed or endorsed by the publisher.

## Footnotes

^1^http://infuse.ukdataservice.ac.uk

^2^https://www.ethnicity-facts-figures.service.gov.uk/uk-population-by-ethnicity/national-and-regional-populations/regional-ethnic-diversity/latest#area-of-england-and-wales-by-ethnicity

^3^https://assets.publishing.service.gov.uk/government/uploads/system/uploads/attachment_data/file/465791/English_Statistical_Release.pdf

^4^https://cks.nice.org.uk

^5^https://www.gov.uk/government/statistics/english-indices-of-deprivation-2019

^6^npda-results.rcpch.ac.uk
